# Increasing Frequencies of Antibiotic Resistant Non-typhoidal *Salmonella* Infections in Michigan and Risk Factors for Disease

**DOI:** 10.3389/fmed.2019.00250

**Published:** 2019-11-08

**Authors:** Sanjana Mukherjee, Chase M. Anderson, Rebekah E. Mosci, Duane W. Newton, Paul Lephart, Hossein Salimnia, Walid Khalife, James. T. Rudrik, Shannon D. Manning

**Affiliations:** ^1^Department of Microbiology and Molecular Genetics, Michigan State University, East Lansing, MI, United States; ^2^Clinical Microbiology Laboratory, University of Michigan, Ann Arbor, MI, United States; ^3^Microbiology Division, Detroit Medical Center University Laboratories, Wayne State University, Detroit, MI, United States; ^4^Microbiology, Immunology & Molecular Laboratories, Sparrow Hospital, Lansing, MI, United States; ^5^Bureau of Laboratories, Michigan Department of Health and Human Services, Lansing, MI, United States

**Keywords:** non-typhoidal *Salmonella*, antimicrobial resistance, epidemiology, risk factors, Michigan

## Abstract

Non-typhoidal *Salmonella* (NTS) are important enteric pathogens causing over 1 million foodborne illnesses in the U.S. annually. The widespread emergence of antibiotic resistance in NTS isolates has limited the availability of antibiotics that can be used for therapy. Since Michigan is not part of the FoodNet surveillance system, few studies have quantified antibiotic resistance frequencies and identified risk factors for NTS infections in the state. We obtained 198 clinical NTS isolates via active surveillance at four Michigan hospitals from 2011 to 2014 for classification of serovars and susceptibility to 24 antibiotics using broth microdilution. The 198 isolates belonged to 35 different serovars with Enteritidis (36.9%) predominating followed by Typhimurium (19.5%) and Newport (9.7%), though the proportion of each varied by year, residence, and season. The number of Enteritidis and Typhimurium cases was higher in the summer, while Enteritidis cases were significantly more common among urban vs. rural residents. A total of 30 (15.2%) NTS isolates were resistant to ≥1 antibiotic and 15 (7.5%) were resistant to ≥3 antimicrobial classes; a significantly greater proportion of Typhimurium isolates were resistant compared to Enteritidis isolates and an increasing trend in the frequency of tetracycline resistance and multidrug resistance was observed over the 4-year period. Resistant infections were associated with longer hospital stays as the mean stay was 5.9 days for patients with resistant isolates relative to 4.0 days for patients infected with susceptible isolates. Multinomial logistic regression indicated that infection with serovars other than Enteritidis [Odds ratio (OR): 3.8, 95% confidence interval (CI): 1.23–11.82] as well as infection during the fall (OR: 3.0; 95% CI: 1.22–7.60) were independently associated with resistance. Together, these findings demonstrate the importance of surveillance, monitoring resistance frequencies, and identifying risk factors that can aid in the development of new prevention strategies.

## Introduction

The gram-negative pathogen, *Salmonella enterica*, is an important public health concern resulting in 93.8 million cases of foodborne infections globally each year (1). In 2015, infection with non-typhoidal *S. enterica* (NTS) serovars was a leading cause of death, with 90,300 deaths reported (2). Infections with NTS can cause nausea, vomiting, abdominal pain, myalgia (muscle pain) and arthralgia (joint pain), while hepatomegaly (liver enlargement), and splenomegaly (spleen enlargement) can develop in some cases (3). Systemic infections can also occur in immunocompromised patients (4). Because of these complications, NTS infections were estimated to contribute to 70 disability-adjusted life years (DALY) lost/100,000 persons worldwide in 2010 (5). In the U.S., the number of NTS infections was estimated to be 1.2 million per year with 23,000 hospitalizations and 450 deaths (6). *Salmonella* infections were also reported to have the highest mean cost of illness among all foodborne infections (7). Geographical differences in serovar prevalence have been documented as well (8). In Europe and Asia, for instance, *S*. Enteritidis was the leading cause of clinical infections in 2002, whereas *S*. Typhimurium was the highest in North America followed by *S*. Enteritidis, *S*. Newport and *S*. Heidelberg. The Typhimurium and Enteritidis serovars, however, have been reported to cause the greatest number of enterocolitis and bacteremia cases (3).

NTS has been frequently isolated from commercially raised chickens and other poultry, and contact with cattle, pigs, horses, and other domestic animals are important risk factors for NTS infections (9). In addition, 74,000 *Salmonella* infections have been attributed to reptile and amphibian exposures in the U.S. each year (10); contact with reptiles and cats was associated with salmonellosis in a prior Michigan study (11). Other studies have identified serovar-specific risk factors as well. A study in the Netherlands, for example, found consumption of raw eggs and products containing raw eggs to be linked to *Salmonella* Enteritidis infections, while exposure to raw meat and playing in a sandbox were risk factors for *S*. Typhimurium infections (12). Prior history of antibiotic use, living on a livestock farm, and international travel were also identified as risk factors for *S*. Typhimurium infections in Canada (13). NTS isolates have been recovered from environmental sources including water and soil and can often survive in these environments for extended periods of time (14, 15). Taken together, these studies indicate the importance of the environment as a reservoir for *Salmonella*.

Antibiotic-resistant NTS infections have also emerged and are increasing in frequency in the U.S. resulting in high hospitalization rates and ~$365,000,000 in medical costs every year (16). Fluoroquinolones, third generation cephalosporins, penicillins, macrolides, and trimethoprim-sulfamethoxazole are commonly prescribed for the treatment of salmonellosis, particularly in patients with immunocompromising conditions, young children and the elderly (17). Importantly, antibiotic resistant *Salmonella* infections have been linked to more severe disease outcomes including bloodstream infections as well as hospitalization (18) and multidrug resistance has emerged. Resistance to ampicillin, chloramphenicol, streptomycin, sulfonamides and tetracycline, previously defined as ACSSuT isolates, for instance, has been reported in multiple NTS serovars in different geographical regions (19–22). In addition to ACSSuT, resistance to ampicillin, streptomycin, sulfonamides, and tetracycline (ASSuT) has been observed in *S*. Typhimurium from humans, food animals and retail meats in the U.S. (23). The emergence of these multi-drug phenotypes in *Salmonella* are of great concern since alternative antibiotics that can be used to treat *Salmonella* infections are limited.

Emergence of widespread resistance in *Salmonella* is attributed to the overuse of antibiotics and limits the effectiveness of these antibiotics for the treatment of human infections. Michigan is not included in the CDC FoodNet surveillance network, which monitors the incidence of foodborne illnesses and collects case information from 10 states in the U.S., covering 15% of the U.S. population. Consequently, we sought to examine the distribution of NTS serovars causing disease in Michigan and evaluate risk factors for infection using an active surveillance system at four hospitals. We also calculated the frequency of antibiotic resistance in NTS isolates and identified factors associated with resistance and NTS infections. This study highlights the importance of enhanced surveillance for resistant pathogens to ensure that the most appropriate drug targets are used, and to identify risk factors for infection and patients with an increased risk of more debilitating conditions.

## Materials and Methods

### Strain Source and Collection

From 2011 to 2014, 198 NTS isolates were collected as part of the Enterics Research Investigational Network (ERIN), which was set up in collaboration with the Michigan Department of Health and Human Services (MDHHS) and four major hospitals in southern Michigan. Serovar classification was conducted at the MDHHS using traditional serotyping protocols as recommended by the Association of Public Health Laboratories (24); serovar data was extracted from the Michigan Disease Surveillance System (MDSS) for each case. To ensure that the ERIN surveillance network was representative of enteric infections occurring in Michigan, the frequency of ERIN cases was shown to be similar to those identified throughout the state of Michigan during the same time period (25). Isolates were stored in Luria-Bertani broth with 10% glycerol at −80°C until further testing.

### Antimicrobial Susceptibility Profiling

For NTS, susceptibilities to 24 antibiotics were determined by broth microdilution using Sensititre GN4F Trek plates (Trek Diagnostic Systems, Cleveland, OH, USA) according to the manufacturer's instructions. Nine antibiotic classes were tested and included: aminoglycosides (amikacin, gentamicin, tobramycin); β-lactam antibiotics [penicillins (ampicillin, piperacillin); β-lactam/β-lactamase inhibitor combinations (ampicillin/sulbactam 2:1 ratio, piperacillin/ tazobactam constant 4, ticarcillin/clavulanic acid constant 2); cephalosporins (cefazolin, ceftazidime, ceftriaxone, cefipime)]; carbapenems (imipenem, doripenem, ertapenem, meropenem); tetracyclines (tetracycline, minocycline); fluoroquinolones (ciprofloxacin, levofloxacin); glycylcyclines (tigecycline); nitrofurans (nitrofurantoin); monobactams (aztreonam); and anti-folates (trimethoprim/sulfamethoxazole). The minimum inhibitory concentration (MIC) was determined by identifying the lowest concentration of antibiotic that prevented visible bacterial growth. *Escherichia coli* ATCC 25922, which is susceptible to all antibiotics evaluated, was used as the quality control strain. The results of the susceptibility tests were interpreted as resistant or susceptible in accordance with published guidelines (26) and isolates were classified as multidrug resistant if they were resistant to three or more antimicrobial classes.

### Data Analysis

Epidemiological, demographic, and clinical data were obtained from the MDSS and managed using Microsoft Access and Excel. Season was classified as spring (March, April, and May), summer (June, July, and August), fall (September, October, and November) and winter (December, January, and February) based on the sample collection date; for those cases with a missing collection date, the stool arrival date and/or onset dates were used. Counties in Michigan were classified as urban or rural based on the classification scheme devised by the National Center for Health Statistics; only 10 Michigan counties were designated as urban (27). Based on the published rates of antibiotic prescription use in adults and children in Michigan (28), counties were classified as having high or low prescribing rates. High rates were defined as those counties where hospital service areas had >30% higher use rates relative to the state average. A dichotomous variable was created for length of hospital stay by defining a long hospital stay as one that was greater than 4 days.

SAS version 9.4 (SAS Institute, Cary, NC, USA) and Epi Info™ version 7.0 were used for all statistical analyses. χ^2^ test and Fisher's exact test were used for dichotomous variables to identify significant associations between the dependent and independent variables; a *p*-value ≤ 0.05 was considered significant. A univariate analysis was first conducted, and those variables found to have strong associations with the outcome (*p*-value ≤ 0.20) were included in the multivariate analysis unless the variable contained >15 missing values. Multivariate analysis using forward logistic regression was performed to build a model containing significant variables (*p*-value ≤ 0.05) along with potentially confounding factors such as age and sex. The Mantel-Haenszel χ^2^ test was used to test for trends and the student's *t*-test was used for testing statistical significance between means. Finally, the χ^2^ test for equality of proportions was used for comparing sample proportions.

## Results

### Characteristics of Cases With Non-typhoidal *Salmonella* (NTS) Infections

A total of 198 NTS cases were identified and roughly half were male (53.1%; *n* = 104) and between 18 and 52 years of age (*n* = 81; 40.9%). Forty-four (22.2%) cases were younger than 10 years of age and 14 were younger than 2 years. More cases self-reported as Caucasians (*n* = 125; 74.0%) than as African Americans (*n* = 33; 19.5%) or other races (*n* = 11; 6.5%); race was not known for 29 cases. Diarrhea (*n* = 172, 97.2%), abdominal pain (*n* = 135, 80.4%), and fever (*n* = 106, 69.3%) were commonly reported symptoms, though 70 (41.9%) patients also reported bloody diarrhea. Sixty-five patients (34.6%) were hospitalized between 1 day and 17 days; the average duration of hospitalization was 4.4 days.

No significant difference in the proportion of cases was observed by year (*p-*value = 0.075), though a higher number was reported in the summer and fall (*n* = 139; 70.2%) compared to the winter and spring months (*p-*value < 0.0001). Differences were also observed among the four participating hospitals, which represented 10.6, 23.2, 29.3, and 36.9% of the 198 cases. When stratified by county, 56.2% (*n* = 108) of the cases lived in rural counties and 43.7% (*n* = 84) resided in urban counties; the residence was not known for one case. A subset of five cases resided in other states (Colorado, Georgia, Illinois, Ohio, and South Dakota), though each patient developed symptoms and were diagnosed with salmonellosis while visiting Michigan. Of the 166 cases with travel information available, a significantly higher proportion of patients had not traveled in the past month (*n* = 103, 62.0%) when compared to those who reported travel (*n* = 63; 37.9%) (*p-*value = 0.0019).

Patients reporting contact with animals (*n* = 95; 61.3%) were also more likely to be affected when compared to those reporting no animal contact (*n* = 60; 38.7%) (*p-*value = 0.0049). Among these 95 cases, 12 (12.6%) had contact with reptiles such as turtles and lizards, and nine (9.5%) reported contact with livestock including cattle, horses, goats, or pigs. Contact with domestic animals (e.g., cats, dogs, and small mammals) was also reported in 83 of the 95 (87.4%) cases.

Because more cases resided in rural counties, we conducted a case-case analysis between the rural and urban cases to further detect differences in disease frequencies (Table S1); the five cases from other states were excluded from this analysis. Notably, any animal contact was more common in patients living in rural vs. urban counties (OR: 1.8; 95% CI: 0.95–3.57), however, the association was not statistically significant. Contact with “other” animals including small mammals, fish and/or amphibians was also more common in rural cases (Fisher's exact *p*-value = 0.0095), though only one case from the urban counties reported “other” contact. No significant difference was observed by season or for well water consumption in rural (*n* = 18, 19.8%) vs. urban (*n* = 7, 11.9%) cases; however, pork consumption was significantly lower (OR: 0.1; 95% CI: 0.02–1.12) and peanut butter consumption was significantly higher (OR: 2.1, 95% CI: 1.06–4.35) in rural vs. urban cases.

### Risk Factors for More Severe NTS Infections

To identify predictors of hospitalization, a marker for more severe infections, we used hospitalization as the dependent variable among the 165 cases with data available. In the univariate analysis, patients self-reporting nausea (OR: 2.0; 95% CI: 1.05–3.91) and vomiting (OR: 1.9; 95% CI: 0.99–3.64) were significantly more likely to be hospitalized as were patients over the age of 59 years (*n* = 16; 51.6%). Patients from urban counties were also more likely to be hospitalized (*n* = 34; 41.9%) than cases from rural counties (*n* = 29; 27.9%). No associations were identified with specific serovars.

Multivariate logistic regression using forward selection identified urban residence (OR: 2.1; 95% CI: 1.06–4.04) and vomiting (OR: 1.9; 95% CI: 0.99–3.78) to be the strongest predictors of hospitalization while controlling for age and sex. Because vomiting and nausea were associated with each other (*p-value* < 0.0001), only vomiting was included in the model. Age over 59 years met the *p* > 0.25 significance level for entry into the model but despite the positive association (OR: 2.0; 95% CI: 0.87–4.78), it was not statistically significant.

### Distribution of *Salmonella enterica* Serovars in Michigan Cases

The 198 NTS isolates were classified into 35 different *S. enterica* serovars; the serovar could not be determined for three isolates (Table S2). Among the 195 typed isolates, the predominant serovar was Enteritidis (*n* = 72; 36.9%) followed by Typhimurium (*n* = 38; 19.5%), Newport (*n* = 19; 9.7%), Hartford (*n* = 6; 3.1%), Saintpaul (*n* = 5; 2.6%), and Heidelberg (*n* = 4; 2.1%). The remaining 51 isolates represented 28 different serovars with fewer than three isolates per type. Nine isolates were classified as I 4, [5], 12:i:- /I 4, 5,12:i- (*n* = 3), I 4, 12:b- (*n* = 3), I 4, 12:i:- (*n* = 3), and III 50:Kz (*n* = 1), which likely represent variants of known serovars.

A significant difference in the proportion of Enteritidis cases was observed by hospital (*p-*value = 0.02). The highest proportion was observed at two sites with 24 (33.3%) and 25 (34.7%) Enteritidis cases, though 12 (16.7%) and 11 (15.3%) additional cases were identified at the remaining two sites. The proportion of Typhimurium cases also differed at the four hospitals (*p*-value = 0.003) with frequencies of 44.7% (*n* = 17), 34.2% (*n* = 13), 13.2% (*n* = 5), and 7.9% (*n* = 3) per site. *S*. Heidelberg was recovered from only one case at each hospital, but significant differences were observed in the proportion of Newport cases across hospitals (*p-*value = 0.009) as well as cases with all other serovars combined (*p-*value = 0.0003).

Differences were also observed in the proportion of specific serovars by year. In 2011 and 2012, for instance, 21.1% (*n* = 12) of the 57 cases and 35.2% (*n* = 18) of the 51 cases, respectively, were classified as Enteritidis, whereas higher frequencies were observed in 2013 (*n* = 20 of 34; 58.8%) and 2014 (*n* = 22 of 53; 41.5%). The proportion of Typhimurium cases was similar in years 2011, 2012, and 2014 (average = 16.2%), though a greater proportion of cases (*n* = 12; 35.3%) were recovered in 2013. Of the 19 Newport cases, the proportion of Newport cases decreased in 2013 (*n* = 2; 10.5%) and 2014 (*n* = 3; 15.8%) relative to 2011(*n* = 7; 36.8%) and 2012 (*n* = 7; 36.8%), however, the difference between each period was not significant (*p*-value = 0.22). Only four Heidelberg cases were identified and three of these cases were from 2014. Among all other serovars reported, it is notable that the highest frequencies were recovered in 2011 (*n* = 28; 49.1%) followed by 2014 (*n* = 17; 32.1%) and 2012 (*n* = 17; 33.3%). A more diverse serovar population was reported in 2011 as well given that infections were caused by 26 different serovars, while 15 and 17 serovars were reported in 2012 and 2014, respectively. Only Enteritidis, Typhimurium and Newport were recovered in 2013.

**Figure 1:**
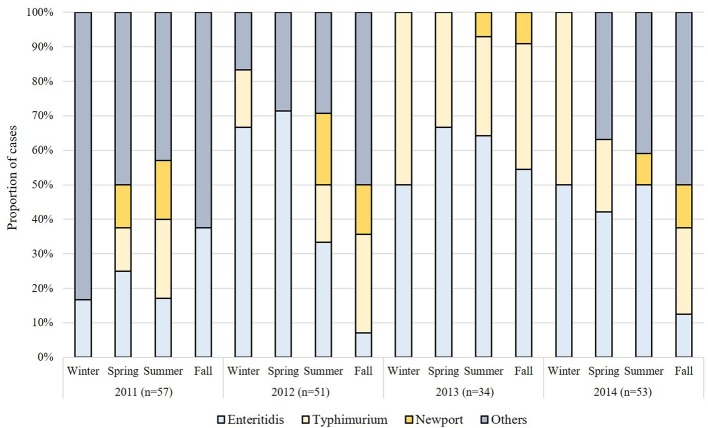
Seasonal variation in the distribution of non-typhoidal *Salmonella* (NTS) serovars in Michigan by year.

Because the number of cases differed by season with the highest proportion of cases occurring in summer than in other seasons (*p*-value < 0.0001), we also examined seasonal variation by serovar (Figure 1). The number of Enteritidis cases was significantly higher in the summer months (*n* = 34; 47.2%) when compared to the fall (*n* = 11; 15.3%), winter (*n* = 10; 13.9%), and spring (*n* = 17; 23.6%) months (*p*-value = 0.0001). Similar trends were observed for Typhimurium in the summer (*n* = 16, 42.1%) vs. fall (*n* = 10; 26.3%), winter (*n* = 6; 15.8%), and spring (*n* = 6; 15.8%) months. The majority (*n* = 14; 73.7%) of the 19 Newport cases were reported in the summer as were most cases infected with other serovars (*n* = 31; 46.9%). During the summer months, the greatest proportion of cases were caused by Enteritidis (*n* = 34, 35.8%), Typhimurium (*n* = 16, 16.8%), and Newport (*n* = 14, 14.7%).

### Risk Factors for Infection With Specific *Salmonella* Serovars

Several serovar-specific factors were identified. In the univariate analysis, significantly more Enteritidis cases were reported in urban (*n* = 40; 57.9%) vs. rural counties (*n* = 31; 37.3%) (*p*-value = 0.011). Enteritidis was also associated with consumption of bottled water at home (*n* = 11; 64.7%) relative to municipal (*n* = 30; 37.0%) (*p*-value = 0.03) or well water (*n* = 10, 52.6%) (*p*-value = 0.21) as well as pork consumption (OR: 7.2; 95% CI: 0.85–61.5). The small sample sizes and missing data records for these variables, however, may have skewed the associations and prevented their inclusion into the multinomial regression analysis.

By contrast, Typhimurium infections were more common in the 95 patients reporting animal contact (*n* = 24; 25.3%) than those without animal contact (*n* = 7; 12.1%) (*p*-value = 0.078). Contact with livestock (Fisher's exact *p*-value = 0.0002) and other animals such as amphibians, small mammals, and fish (OR: 3.8; 95% CI: 1.41–9.97) were more common in Typhimurium cases relative to cases infected with other serovars.

A multinomial logit model using forward selection was used containing the following three outcomes: (1) Enteritidis infection; (2) Typhimurium infection; and (3) infection by all other serovars. When controlling for age and sex, residence was found to be a significant predictor of Enteritidis but not Typhimurium infections. Specifically, cases living in rural areas were significantly less likely to have infections caused by Enteritidis (OR: 0.4; 95% CI: 0.23–0.85; *p*-value = 0.01) but not Typhimurium (OR: 1.2; 95% CI: 0.52–2.71; *p*-value = 0.69); only the former association was significant. Because of missing data and too few cases per outcome, however, no additional variables could be examined in the multivariate analysis.

### Antibiotic Resistance Profiles in 198 Non-typhoidal *Salmonella* (NTS) Isolates

**Figure 2:**
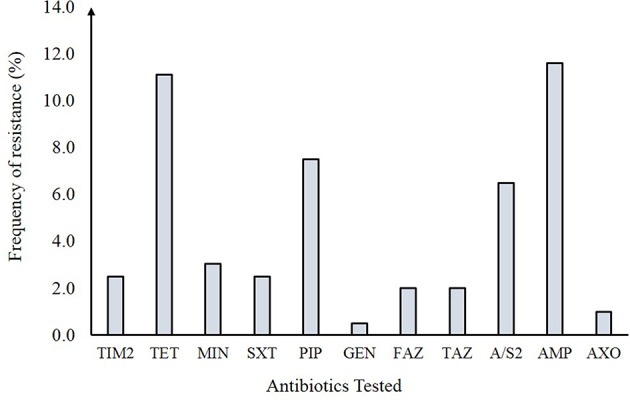
Antibiotic resistance frequencies in 198 non-typhoidal *Salmonella* (NTS) isolates from Michigan. TIM2, Ticarcillin / clavulanic acid constant 2; TET, Tetracycline; MIN, Minocycline; SXT, Trimethoprim / sulfamethoxazole; PIP, Piperacillin; \ GEN, Gentamicin; FAZ, Cefazolin; TAZ, Ceftazidime; A/S2, Ampicillin / sulbactam 2:1 ratio; AMP, Ampicillin; AXO, Ceftriaxone.

**Figure 3:**
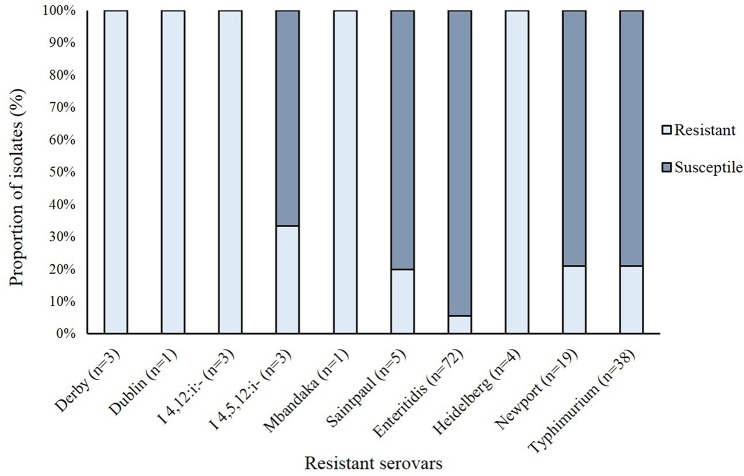
Frequency of antibiotic resistance in 149 non-typhoidal *Salmonella* (NTS) isolates representing the 10 serovars with at least one resistant isolate.

Resistance to at least one antibiotic was observed among 30 of the 198 (15.1%) NTS isolates (Figure 2). Resistance to the β-lactam, ampicillin (11.6%), and tetracycline (11.1%) predominated followed by resistance to trimethoprim-sulfamethoxazole (2.5%), gentamicin (0.5%), and other β-lactams including cephalosporins like cefazolin (2.0%), ceftazidime (2.0%), and ceftriaxone (1.0%). No resistance was observed to 13 of the 24 antibiotics tested. Overall, 19 distinct antibiotic resistance patterns were observed among the 30 resistant NTS isolates (Table S3). Multidrug resistance (MDR) to ≥ 3 antimicrobial classes was observed in 15 (7.5%) isolates while four (2.0%) isolates were resistant to ≥ 4 antimicrobial classes; nine (4.5%) isolates were resistant to only one antimicrobial class. When stratified by serovar (Figure 3), only 10 of the 35 NTS serovars contained isolates that were resistant to at least one antibiotic. Among these 10 serovars, Enteritidis was significantly less likely to comprise resistant isolates than Typhimurium (Fisher's exact *p*-value = 0.022) as well as the other eight serovars combined (Fisher's exact *p*-value < 0.0001). In all, four *S*. Enteritidis (*n* = 72; 5.6%) and eight *S*. Typhimurium (*n* = 38; 21.0%) isolates were resistant to at least one antibiotic.

**Figure 4:**
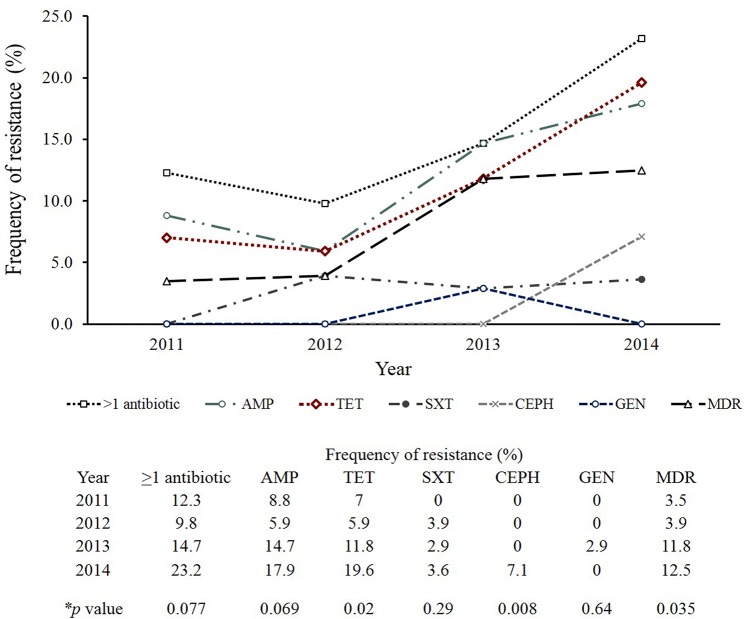
Trends in antibiotic resistance among clinical non-typhoidal *Salmonella* (NTS) isolates from Michigan over time. Mantel-Haenszel chi-square was used to identify trends over time and calculate *p*-values. AMP, Ampicillin; TET, Tetracycline; SXT, Trimethoprim/sulfamethoxazole; CEPH, Cephalosporin; GEN, Gentamicin; MDR, Multidrug resistance (resistance to ≥3 antimicrobial classes).

**Figure 5:**
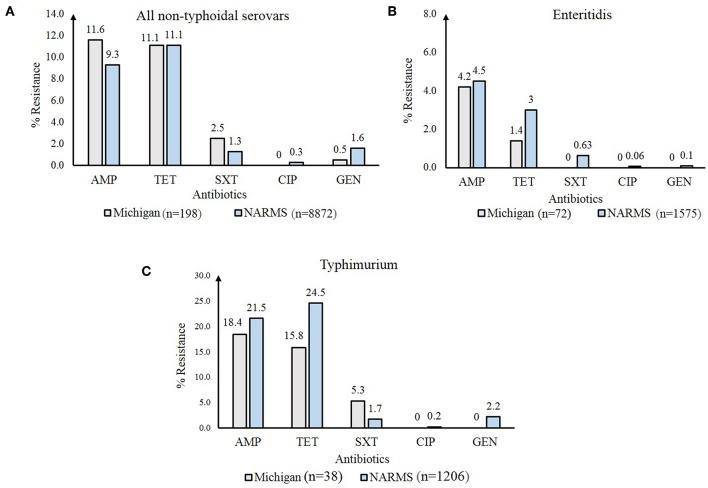
Frequency of antibiotic resistance among Non-Typhoidal *Salmonella* (NTS) isolates from Michigan compared to those from the National Antimicrobial Resistance Monitoring System (NARMS) (29), 2011-2014. Resistance frequencies in: **(A)** all NTS serovars; **(B)** Enteritidis; and **(C)** Typhimurium. AMP, ampicillin; TET, tetracycline; SXT, trimethoprim-sulfamethoxazole; CIP, ciprofloxacin; GEN, gentamicin.

Important differences in resistance frequencies were also identified by year of isolation (Figure 4). Significant increases in resistance to tetracycline and cephalosporins, for example, were observed between 2011 and 2014 as were increases in multidrug resistance (*p*-value < 0.05). No significant difference in the frequency of resistance to trimethoprim-sulfamethoxazole or gentamicin were noted. A comparison between all NTS isolates from Michigan to those tested by the National Antimicrobial Resistance Monitoring System (NARMS) (29) during the same time period also revealed slight differences in resistance frequencies by antibiotic (Figure 5A). None of these differences, however, were significant. For Enteritidis, tetracycline resistance was lower in Michigan (*n* = 1, 1.4%) than NARMS (*n* = 48, 3.0%) isolates (Figure 5B), while resistance to ampicillin and tetracycline was higher in Typhimurium isolates from NARMS than Michigan (Figure 5C). Resistance to trimethoprim-sulfamethoxazole was also higher in the Michigan Typhimurium isolates (*n* = 2, 5.3%) compared to the NARMS isolates (*n* = 21, 1.7%), yet none of the frequency differences observed among Enteritidis and Typhimurium isolates were statistically significant.

### Epidemiological Associations With Antibiotic Resistant NTS Infections

To identify factors associated with resistant NTS infections, we conducted univariate and multivariate analyses using resistance to at least one (≥ 1) antibiotic as the dependent variable. The univariate analysis demonstrated that the odds of resistance was significantly higher in Typhimurium isolates (OR: 4.5; 95% CI: 1.27-16.22) and other NTS serovars (OR: 4.6; 95% CI: 1.47-14.20) compared to Enteritidis isolates (Table 1). Higher resistance frequencies were also observed in counties with low antibiotic prescribing rates (*n* = 26, 16.9%) compared to those with high rates (*n* = 4, 10.3%), yet this difference was not statistically significant. Furthermore, frequencies of antibiotic resistant infections were higher in urban (*n* = 14, 16.7%) than rural (*n* = 16, 14.8%) areas and were lowest in the summer (*n* = 10, 10.3%) compared to winter, spring and fall (*n* = 20, 19.8%) (OR: 2.1; 95% CI: 0.95-4.86).

Table 1Univariate and multivariate analysis to identify factors associated with antibiotic resistance in 198 clinical non-typhoidal *Salmonella* in Michigan, 2011–2014.

**Table:** 

**Characteristic**	Total isolates^*^	**No (%) ≥1 resistance**	**OR (95% CI^†^)**	***p-*value^‡^**
**PATHOGEN FACTORS**				
**Serovar**				
Enteritidis	72	4 (5.6%)	–	0.008
Typhimurium	38	8 (21.1%)	–	
Other	85	18 (21.2%)	–	
**Outbreak associated**				
Yes	7	3 (42.9%)	–	0.06
No	39	4 (10.3%)	–	
**DEMOGRAPHIC FACTORS**				
**Residence**				
Urban	84	14 (16.7%)	1.1 (0.53-2.51)	0.72
Rural	108	16 (14.8%)	1.0	
**Age in years**				
0–10	44	7 (15.9%)	1.3 (0.35-4.97)	0.75
>10–59	122	19 (15.6%)	1.3 (0.41-4.10)	0.79
>59	32	4 (12.5%)	1.0	
**Sex**				
Male	104	17 (16.3%)	1.0	0.51
Female	92	12 (13.0%)	0.8 (0.34–1.71)	
**Race**				
Caucasian	125	19 (15.2%)	–	0.62
Other	44	5 (11.4%)	–	
**Antibiotic prescription rates by county**				
High	39	4 (10.3%)	–	0.46
Low	153	26 (16.9%)	–	
**EPIDEMIOLOGICAL AND OTHER FACTORS**				
**Length of hospital stay**				
No hospitalization	123	17 (13.8%)	1.0	
1–4 days	36	5 (13.9%)	–	1.0
≥5 days	23	6 (26.1%)	2.2 (0.76-6.37)	0.14
**Season**				
Fall	42	12 (28.6%)	3.5 (1.36-8.80)	0.0067
Winter	22	2 (9.1%)		1.0
Spring	37	6 (16.2%)	1.7 (0.57-5.03)	0.35
Summer	97	10 (10.3%)	1.0	
**Domestic travel in the past month**				
Yes	46	9 (19.6%)	1.7 (0.69-4.22)	0.25
No	120	15 (12.5%)	1.0	
**Animal contact**				
Yes	95	11 (11.6%)	0.6 (0.23-1.44)	0.24
No	60	11 (18.3%)	1.0	
**Chicken consumption**				
Yes	111	14 (12.6%)	0.7 (0.25–1.98)	0.50
No	87	6 (17.1%)	1.0	
**Water at home**				
Any municipal	103	14 (13.6%)	1.0	0.53
Any well	25	5 (20.0%)	1.6 (0.51-4.92)	0.52
Only bottled	22	4 (18.2%)	1.4 (0.42-4.79)

**Table:** 

**Characteristic**	**Multivariate Analysis**		
	**OR**	**95% CI €**	***p*-value^‡^**
Sex: Female	0.7	0.31–1.75	0.76	
Age in years: ≥ 60	0.7	0.20–2.74	0.82	
Serovar: All serovars except Enteritidis	3.8	1.24–11.82	0.02	
Season: Fall	3.0	c1.22–7.60	0.02	
Hospitalization duration: ≥5 days	2.7	0.82–8.65	0.10	

* *Depending on the variable examined, the number of isolates do not add up to the total (n = 198) because of missing data*.

*€ Wald 95% confidence intervals (CI) for odds ratio (OR)*.

‡ *p-value was calculated by Chi square test and Fisher's exact test was used for variables <5 in at least one cells; ORs were not calculated for variables with <5 per cell and the Mantel-Haenszel Chi square test was used for serogroup*.

*£Logistic regression was performed using forward selection while controlling for variables that yielded significant (p-value ≤ 0.05) and strong (p-value ≤ 0.20) associations with in the univariate analysis and with a sufficient sample. Nineteen records were omitted from the analysis when hospitalization was added to the model, however, the associations between resistance and the other four variables remained the same with and without this variable. Hosmer and Lemeshow Goodness of Fit tests were >0.05 for both models with and without hospitalization. All variables were tested for collinearity*.

Notable differences in resistance frequencies were observed among the 59 patients who were hospitalized for one or more days. Among these 59 cases, the mean hospital stay was 5.9 days for the 39 patients hospitalized with resistant NTS isolates compared to 4.0 days for the 48 patients infected with pansusceptible isolates (Table S4). Six of the 23 (26.1%) patients with hospital stays of ≥5 days had resistant infections compared to five of the 36 (13.9%) patients with stays between 1 and 4 days (Student's *t*-test *p*-value < 0.05). Cases with tetracycline resistant and susceptible infections had mean hospital stays of 6.0 days and 4.2 days, respectively (Student's *t*-test *p*-value = 0.068), whereas the mean hospital stay was 6.2 days for patients infected with ampicillin-resistant NTS compared to 4.0 days for patients with ampicillin-susceptible infections (Student's *t*-test *p*-value < 0.05). No association was observed between hospitalization and infection with either Enteritidis (*n* = 21, 30.9%) or Typhimurium (*n* = 11, 31.4%) when compared to all other serovars (*n* = 30, 36.6%).

Multivariate analysis using forward regression was performed to identify predictors of resistant infections. Because the frequency of resistance to ≥1 antibiotic was similar in isolates belonging to Typhimurium and the other NTS serovars except Enteritidis, they were grouped together for the multivariate analysis. After adjusting for sex and age, the model indicated that compared to Enteritidis, the remaining serovars were 3.8 times more likely to be resistant to at least one antibiotic (Table 1). Moreover, resistant NTS infections were significantly more likely to occur in fall (OR: 3.1; 95% CI: 1.22–7.60) than in the other three seasons. A strong association (OR: 2.7) was also observed between resistance and hospitalization stays longer than 5 days, however, it was not statistically significant while controlling for the other four variables.

## Discussion

This study, which was conducted between 2011 and 2014 using data and isolates from an active surveillance system at four large, metropolitan hospital systems, indicated that most (64.1%) of the 198 cases were over the age of 19 and 16.2% were over 60 years of age. These data differ from those reported in a prior Michigan study of Enteritidis infections occurring between 1995 and 2001, which found children <4 years of age to have the greatest risk of infection (30). Although age was not significantly associated with Enteritidis infections or infections caused by any other serovars, these data suggest that the demographics of salmonellosis cases may have changed over time. Additional studies in different Michigan hospitals are therefore required.

Significantly more NTS infections occurred in Michigan during the summer and fall months consistent with a CDC report showing that most *Salmonella* infections occur between June and October in the U.S. (31). An increased frequency of infections caused by *S*. Enteritidis specifically (30) and other enteric pathogens has also been reported during the summer. Shiga toxin-producing *E. coli* (STEC) infections, for example, were highest in Michigan during the summer (37.3%) and fall (29.5%) over a 12-year period (2001–2012) (32). Such seasonal variation has been explained by recreational activities, inadequate cooking and suboptimal food storage temperatures during outdoor picnics and barbeques (33) as well as visiting petting zoos and farms (34). Variation in the distribution of serovars by season was also observed and is consistent with data from a study of 690,479 *Salmonella* infections reported to the CDC. Specifically, this study found that the number of different *Salmonella* serotypes causing human infection, or the “serotype richness,” was greatest in the winter even though the greatest number of infections occurred during the summer (35).

Although salmonellosis frequencies were not significantly higher in patients residing in rural vs. urban areas (*p*-value = 0.083), urban residence was found to be a predictor of infection with *S*. Enteritidis using logistic regression. Intriguingly, urban residence was also associated with hospitalization due to any NTS infection, a finding that may be related to proximity of the health care facilities included in the study. The association between residence location and infection with specific serovars is supported by data from prior studies showing a lower prevalence of Enteritidis in the farm environment relative to other serovars. A Canadian study of urban and rural streams, for example, detected low frequencies of Enteritidis in the rural streams (36), while Enteritidis was rarely recovered from livestock and poultry in Alberta (37) or in the environment of commercial poultry farms in California (38). It is therefore possible that *Salmonella* serovars other than Enteritidis may be more widespread in the environment. An association was also observed between Typhimurium infections and history of animal contact, which has been reported in many prior studies. Indeed, one study indicated that living on a livestock farm was an independent risk factor for acquiring *S*. Typhimurium DT104 infections (13), while another study recovered indistinguishable *S*. Typhimurium DT104 isolates from a child and animals living on the same farm (39). The identification of animal contact as a risk factor for NTS infections in Michigan may be important as it highlights the need for additional studies to investigate this epidemiological association and develop more targeted prevention strategies. Indeed, we were only able to evaluate whether or not any animal contact was reported, which fails to adequately describe the level or duration of contact or even the well-being of the animals.

Importantly, a wide range of antibiotic resistance profiles in the 198 NTS isolates recovered from patients at four Michigan hospitals was observed. High frequencies of resistance to ampicillin and tetracycline were identified with an increasing trend in tetracycline resistance over the 4-year period; tetracycline resistance also contributed to the high frequency of MDR in the NTS isolates examined. It is notable that an increase in tetracycline resistance was not observed by NARMS for the same time period (*p*-value = 0.66) and hence, it is possible that Michigan harbors a unique population of resistant NTS. NARMS also reported an increased frequency of MDR in 2015 (12%) relative to 2008 (9.5%) (29). While tetracycline is not widely recommended for clinical use, antibiotics such as amoxicillin, penicillin, and sulfamethoxazole-trimethoprim are commonly prescribed to adults and children in Michigan (28). The high prescription rates of these antibiotics, among others, is likely to have an impact on resistance frequencies in the state. Unfortunately, actual antibiotic usage data was not available for NTS cases in our study as these data are not collected by the MDHHS during case interviews. We also observed an increasing trend in the frequency of MDR in NTS over the 2011–2014 time period, however, the difference was not significant by year and could be due to our focused surveillance of only four hospitals. It is possible that increases in MDR frequencies over time could be linked to international travel or food imports as suggested previously (40, 41); however, travel was not associated with MDR infections in our study and import risk was not evaluated.

Although tetracyclines are not widely used in human medicine, they are among the most commonly used antibiotics in livestock and poultry worldwide (42). The FDA has estimated that 3,535,701 (kg)^2^ of tetracyclines are used in food-producing animals in the U.S. each year representing 64% of all antibiotics used (43). While the use of tetracyclines in these animals decreased by 40% in 2016–2017 relative to the period between 2009 and 2017 (43), antibiotic residues and resistance determinants can persist in the environment (44) and animal reservoir, be transmitted to humans, and contribute to MDR in both gram-positive and -negative bacteria (45). Indeed, the emergence and spread of resistance between food animals and people has been documented. One historical study, for instance, found identical resistance patterns in *E. coli* isolates from livestock and farming families (46), while others have documented spread through food products and water (47–49). Since we have only examined resistance in clinical NTS isolates, additional studies are needed to quantify resistance frequencies in isolates from other sources to identify those strain types, virulence gene profiles, and resistance phenotypes that pose the highest risk of transmission to and infection in humans. A recent study, for example, documented different antibiotic resistance profiles among isolates from different sources and suggested that multiple sources in the food-chain are responsible for resistant *Salmonella* infections in humans (23).

As such, it is important to consider that the genetic and phenotypic diversity of NTS isolates in different geographical locations may play a role in human infections as specific NTS lineages circulating in Michigan may be more likely to carry resistance determinants. Support for this possibility comes from our finding that NTS serovars had varying resistance profiles. Notably, multivariate logistic regression identified serovar Enteritidis to be significantly less likely to be resistant to at least one antibiotic compared to all other NTS serovars. Serovar-dependent differences in resistance have been observed in NTS isolates in different geographic locations including Spain (50) and Brazil (51). The reason for serovar-specific differences is not clear, however, one study found that ciprofloxacin-resistant *S*. Typhimurium isolates were more competitive during growth *in vitro* with increasing concentrations of ciprofloxacin than the ciprofloxacin-resistant *S*. Enteritidis isolates (52). Other studies have shown that certain NTS serovars such as Kentucky, Typhimurium, and Heidelberg, were more likely to have MDR (53–55), whereas some serovars (e.g., Enteritidis, Montevideo, Infantis, and Mbandaka) were more commonly pansusceptible or were resistant to fewer antibiotics (56, 57). Such differences could be due to the specificity of or ability to take up certain plasmids carrying resistance genes as some plasmids may be more commonly acquired across serovars and other bacterial species (58). Serovars such as Kentucky and Heidelberg have also been shown to have a mutation in the methyl mismatch repair (MMR) system, which may contribute to genetic heterogeneity (59). This genome plasticity has been offered as an explanation for higher frequencies of antibiotic resistance within these strain backgrounds (60). Differences in fitness between serovars, genetic plasticity and dissimilar resistance mechanisms could partly explain the varying frequencies of resistance in NTS serovars worldwide. Although serogrouping has been useful for differentiating NTS isolates for surveillance studies (61), future studies are needed to characterize the isolates using whole genome sequencing to identify bacterial features commonly associated with specific resistance phenotypes and carriage of specific resistance determinants. Having these data could help identify sources of resistant infections and identify whether some strains are epidemiologically linked.

Our study also identified season to be a predictor of resistant NTS infections, with fewer resistant infections occurring in the summer and more in the fall as is consistent with seasonal variation observed in prior studies. A study of fluoroquinolone resistance in *Campylobacter*, for example, observed higher frequencies in the winter and spring compared to the summer. This difference was attributed to higher consumption of poultry products containing resistant bacteria in the winter and more frequent exposure to susceptible *Campylobacter* through other sources in the summer (62). Our prior studies have also observed higher resistance frequencies in *C. jejuni* (63) and STEC (64) recovered from Michigan patients in the winter and/or spring and could be due, in part, to seasonal variation in antibiotic prescription rates (65, 66). Because prescribing rates may vary by the type of infection and geographic location given climate and other factors, additional studies are required to understand the most important factors that impact antibiotic resistance development and trends in Michigan.

While we did not identify antibiotic resistant NTS infections to be a significant predictor of hospitalization, we did observe a strong association between resistance in NTS and a longer duration (≥5 days) of hospitalization. This finding is not surprising given that resistant infections take longer to clear than susceptible infections during antibiotic treatment and is consistent with other studies. Importantly, studies in other pathogens have identified associations between resistance and mortality, hospitalization, hospital stay duration, and the need for surgery (18, 64, 67, 68).

Because NTS pathogens are a leading cause of enteric infections worldwide and have been shown to frequently resist clinically important antibiotics, continuous surveillance, and monitoring is critical. NTS isolation and routine testing for resistance is imperative in order to help medical personnel and public health officials determine and modify the course of treatment for NTS infections. Furthermore, identifying risk factors for NTS and resistant NTS infections may help in the development of disease management policies and antibiotic use standards aimed at curbing the spread of NTS and resistance determinants.

## Data Availability Statement

All datasets generated for this study are included in the article/Supplementary Material.

## Ethics Statement

The studies involving human participants were reviewed and approved by Institutional Review Boards at Michigan State University (MSU; Lansing, MI, USA; IRB #10-736SM), the MDHHS (842-PHALAB), and the four participating hospitals. Written informed consent from the participants' legal guardian/next of kin was not required to participate in this study in accordance with the national legislation and the institutional requirements.

## Author Contributions

SM performed the experiments with CA and RM organized samples and extracted the epidemiological data. SM and SDM managed the data, conducted the analyses, and drafted the manuscript. SM, SDM, JR, DN, HS, PL, and WK designed the study and organized sample collection at each site. All authors contributed and approved the manuscript content.

### Conflict of Interest

The authors declare that the research was conducted in the absence of any commercial or financial relationships that could be construed as a potential conflict of interest.
