# Supplementary material for: Increasing Frequencies of Antibiotic Resistant Non-typhoidal Salmonella Infections in Michigan and Risk Factors for Disease
Source: Front Med (Lausanne). 2019 Nov 8;6:250. doi: 10.3389/fmed.2019.00250 (PMC6857118; doi:10.3389/fmed.2019.00250)
Supplement: Supplementary file 1 [file Table_1.pdf]

Supplemental Data

Table S1. Characteristics of rural (n=108) and urban salmonellosis cases (n=84).

	Rural		Urban		Univariate analysis: rural vs. urban		
**Variables**	**Total cases***	**No. (%)**	**Total cases***	**No. (%)**	**OR**	**95% CI†**	***p* value‡**
**Food consumption**							
Turkey	38	24 (63.2%)	22	18 (81.8%)	0.4	0.11-1.35	0.15
Chicken	88	64 (72.7%)	58	47 (81.0%)	0.6	0.28-1.39	0.25
Beef	59	50 (84.7%)	40	38 (95.0%)	0.3	0.06-1.43	0.19
Pork	55	45 (81.8%)	34	33 (97.1%)	0.1	0.02-1.12	0.04
Deli meat	86	46 (53.5%)	59	37 (62.7%)	0.7	0.35-1.34	0.27
Raw fruits	41	33 (80.5%)	22	19 (86.4%)	0.6	0.15-2.75	0.73
Raw leafy greens	88	63 (71.6%)	58	41 (70.7%)	1.0	0.50-2.17	0.91
Raw vegetables	58	50 (86.2%)	41	38 (92.7%)	0.5	0.12-1.98	0.35
Peanut butter	88	42 (47.7%)	57	17 (29.8%)	2.1	1.06-4.35	0.03
**Animal contact**							
Any animal contact	94	63 (67.0%)	61	32 (52.5%)	1.8	0.95-3.57	0.07
Reptiles	94	8 (8.5%)	61	4 (6.6%)	--	--	0.76
Livestock	94	8 (8.5%)	61	1 (1.6%)	--	--	0.08
Birds	94	16 (17.0%)	61	2 (3.3%)	--	--	0.0095
Domestic animals	94	56 (59.6%)	61	27 (44.3%)	1.8	0.97-3.56	0.06
Other animals	94	20 (21.3%)	61	1 (1.6%)	--	--	0.0002
**Water source at home**							
Well	91	18 (19.8%)	59	7 (11.9%)	1.8	0.71-4.7	0.26

*The number of cases with a given characteristic did not add up to the total (n=198) because of missing data; for some variables, cases reported more than one characteristic.

† 95% confidence interval (CI) for odds ratio (OR)

‡ *p* value was calculated by Chi-square test and Fisher's exact test was used for variables $\leq$5 in at least one cell; ORs and 95% CIs could not be calculated for those variables with fewer than 5 per cell.

Table S2: Frequency of *Salmonella* serovars identified in Michigan, 2011-2014

Serovar	No. of isolates (%)*
Agona	1 (0.5%)
Anatum	1 (0.5%)
Berta	1 (0.5%)
Braenderup	3 (1.5%)
Derby	3 (1.5%)
Dublin	1 (0.5%)
Enteritidis	72 (36.9%)
Fluntern	1 (0.5%)
Hartford	6 (3.1%)
Heidelberg	4 (2.0%)
Holcomb	1 (0.5%)
I 4, [5], 12:i:- /I 4, 5,12:i-	3 (1.5%)
I 4, 12:b-	3 (1.5%)
I 4, 12:i:-	3 (1.5%)
III 50:Kz	1 (0.5%)
Infantis	3 (1.5%)
Jangwani	1 (0.5%)
Javiana	3 (1.5%)
Kiambu	1 (0.5%)
Mbandaka	1 (0.5%)
Mississippi	1 (0.5%)
Montevideo	3 (1.5%)
Muenchen	1 (0.5%)
Newport	19 (9.7%)
Oranienburg	3 (1.5%)
Panama	2 (1.0%)
Pomona	1 (0.5%)
Poona	1 (0.5%)
Saintpaul	5 (2.6%)
Sandiego	2 (1.0%)
Schwarzengrund	1 (0.5%)
Stanley	2 (1.0%)
Thompson	2 (1.0%)
Typhimurium	38 (19.5%)
Virchow	1 (0.5%)

***** The total does not add up to 198 due to missing serovar data for three isolates.

Table S3. Antibiotic resistance profiles among resistant clinical non-typhoidal *Salmonella* isolates (n=30) in Michigan, 2011-2014.

Antibiotic Resistance Pattern	Number and Proportion of Resistant Isolates (%)
Ampicillin only	2 (6.7%)
Tetracycline only	5 (16.7%)
Trimethoprim-Sulfamethoxazole	1 (3.3%)
Tetracycline, Piperacillin, Ampicillin, Ampicillin-Sulbactam	5 (16.7%)
Piperacillin, Ampicillin, Ampicillin-Sulbactam	3 (10.0%)
Tetracycline, Minocycline	1 (3.3%)
Trimethoprim-Sulfamethoxazole, Piperacillin, Ampicillin, Ampicillin-Sulbactam	1 (3.3%)
Tetracycline, Trimethoprim-Sulfamethoxazole, Ampicillin	1 (3.3%)
Ticarcillin-Clavulanate, Tetracycline, Minocycline, Piperacillin, Ampicillin-Sulbactam, Ampicillin	1 (3.3%)
Ticarcillin-Clavulanate, Tetracycline, Piperacillin, Cefazolin, Ceftazidime, Ceftriaxone, Ampicillin-Sulbactam, Ampicillin	1 (3.3%)
Tetracycline, Minocycline, Piperacillin, Ampicillin	1 (3.3%)
Ticarcillin-Clavulanate, Cefazolin, Ceftazidime, Ampicillin	1 (3.3%)
Tetracycline, Minocycline, Ampicillin-Sulbactam, Ampicillin	1 (3.3%)
Ticarcillin-Clavulanate, Tetracycline, Piperacillin, Cefazolin, Ceftazidime, Ceftriaxone, Ampicillin	1 (3.3%)
Tetracycline, Piperacillin, Ampicillin	1 (3.3%)
Tetracycline, Minocycline, Ampicillin	1 (3.3%)
Tetracycline, Cefazolin, Ceftazidime, Ampicillin	1 (3.3%)
Ticarcillin-Clavulanate, Tetracycline, Minocycline, Trimethoprim-Sulfamethoxazole, Piperacillin, Ampicillin-Sulbactam, Ampicillin	1 (3.3%)
Tetracycline, Trimethoprim-Sulfamethoxazole, Gentamicin, Ampicillin	1 (3.3%)

Table S4. Characteristic of cases with resistant and susceptible NTS infections in Michigan, 2011-2014

Variable	No. (%) cases*	No (%) TET^R	No (%) TET^S	*p* value‡	No (%) AMP^R	No (%) AMP^S	*p* value‡
		Tetracycline			Ampicillin		
Rural residence	108 (54.8%)	13 (12.0%)	95 (87.9%)	0.77	13 (12.0%)	95 (87.9%)	0.97
Animal contact	95 (61.3%)	9 (9.5%)	86 (90.5%)	0.91	9 (9.5%)	86 (90.5%)	0.29
International travel (past month)	20 (12.0%)	2 (10.0%)	18 (90.0%)	1.0	3 (15.0%)	17 (85.0%)	0.71
Domestic travel (past month)	46 (27.7%)	6 (13.0%)	40 (86.9%)	0.46	5 (10.9%)	41 (89.1%)	1.0
Hospitalization	65 (34.6%)	7 (10.8%)	58 (89.2%)	0.89	10 (15.4%)	55 (84.6%)	0.25
Mean days in hospital	4 (n=59)	6 (n=7)	4.15 (n=52)	0.068†	6.2 (n=10)	4 (n=49)	0.0107†
Abdominal pain	135 (80.4%)	15 (11.1%)	120 (88.9%)	0.53	14 (10.4%)	121 (89.6%)	0.21
Body ache	59 (35.5%)	5 (8.5%)	54 (91.5%)	0.79	6 (10.2%)	53 (89.8%)	0.58
Bloody diarrhea	70 (41.9%)	8 (11.4%)	62 (88.6%)	0.65	8 (11.4%)	62 (88.6%)	0.85
Chills	71 (42.5%)	7 (9.9%)	64 (90.1%)	0.91	8 (11.3%)	63 (88.7%)	0.81
Fatigue	85 (50.6%)	10 (11.8%)	75 (88.2%)	0.66	11 (12.9%)	74 (87.1%)	0.86
Headache	54 (32.3%)	6 (11.1%)	48 (88.9%)	0.92	5 (9.3%)	49 (90.7%)	0.37
Nausea	92 (55.1%)	8 (8.7%)	84 (91.3%)	0.34	10 (10.9%)	82 (89.1%)	0.46
Vomiting	65 (39.2%)	6 (9.2%)	59 (90.8%)	0.59	7 (10.8%)	58 (89.2%)	0.56
Fever	106 (69.3%)	11 (10.4%)	95 (89.6%)	1.0	12 (11.3%)	94 (88.7%)	1.0

*The % frequency reported from total cases and the number of cases that were available for each variable are specified.

‡From Chi-square test or Fisher's exact test († Student's t-test for independent means).
